# Stem Cells in Clinical Trials for Pelvic Floor Disorders: a Systematic Literature Review

**DOI:** 10.1007/s43032-021-00745-6

**Published:** 2021-10-01

**Authors:** Stefano Manodoro, Matteo Frigerio, Marta Barba, Sara Bosio, Luigi Antonio de Vitis, Anna Maria Marconi

**Affiliations:** 1 Division of Obstetrics and Gynecology, San Paolo Hospital Medical School, ASST Santi Paolo E Carlo, Via Antonio di Rudinì 8, 20142 Milan, Italy; 2 Division of Obstetrics and Gynecology, San Gerardo University Hospital, Monza, Italy; 3 University of Milano-Bicocca, Monza, Italy; 4 Department of Health Sciences, University of Milano, Milan, Italy

**Keywords:** Stem cells, Regenerative medicine, Tissue engineering, Stress urinary incontinence, Anal incontinence, Pelvic floor disorders, Systematic review

## Abstract

Pelvic floor disorders (PFDs) include a series of conditions that can be poorly tolerated, negatively affecting the quality of life. Current treatment options show unsatisfactory results and new ones are therefore needed. Stem cell (SC) therapy might be an alternative treatment strategy. This systematic review aims to define the state of art of SC therapy for PFDs in clinical trials, by systematically reviewing the available evidence. A systematic search strategy was conducted up to November 7, 2020, in PubMed, Scopus, Cochrane Library, and ISI Web of Science. Preclinical studies on animal models were not considered. Studies were included when the patients were affected by any PFDs and cells were isolated, cultured, and characterized as SC. The study protocol was registered in PROSPERO (CRD42020216551). A total of 11 prospective clinical studies were included in the final assessment, specifically 7 single-arm studies dealing with SC therapy for stress urinary incontinence and 4 with anal incontinence. Among the latter, there were two prospective, single-arm studies and two randomized controlled trials. No papers concerning the use of SC for prolapse repair were retrieved. Due to the great heterogeneity, data pooling was not possible. Stem cell injection resulted in a safe procedure, with few mild adverse side effects, mostly related to harvesting sites. However, a clear beneficial impact of SC treatment for the treatment of pelvic floor disorders could not be demonstrated. Further larger targeted studies with control arms are needed before any conclusions can be made.

**Supplementary Information:**

The online version contains supplementary material available at 10.1007/s43032-021-00745-6.

## Introduction

Pelvic floor disorders (PFDs) include a series of conditions related to a weakening of the pelvic muscles and/or tears of the endopelvic fascia, usually related to obstetric trauma. The most prevalent PFDs include genital prolapse, stress urinary incontinence, and anal incontinence. As a consequence, related symptoms may involve alteration of vaginal, bowel, lower urinary tract, and sexual well-being. They can be poorly tolerated, negatively affecting the quality of life, impairing social and daily activities, and be the cause of emotional distress and isolation [1]. Management of PFDs traditionally involves pelvic floor rehabilitation and subsequent surgical repair in case of conservative therapy failure [2, 3]. Regenerative medicine might offer an alternative treatment strategy. Stem cells (SCs) represent a promising tool for tissue engineering, in particular for skeletal and connective tissue repair [4]. SCs possess multipotent differentiation capabilities, in addition to the fact that they are harvested from multiple tissues (such as muscular and adipose tissue) and expanded in vitro. Possible applications of stem cells in PFDs include prolapse, stress urinary incontinence, and anal incontinence repair [5–7]. Studies using adult SCs to induce tissue regeneration in animal models of stress urinary incontinence, anal incontinence, and genital prolapse have shown promising results [8–10]. The rationale is promoting muscle and nerve regeneration by fusing SCs with existing muscle and releasing trophic factors, such as interleukins and growth factors, that regulate multiple fundamental cellular functions, including proliferation, differentiation, migration, adhesion, and apoptosis [11]. Animal models indicate the feasibility of using autologous cells for functional restoration of urethral sphincter deficiency. For the treatment of stress urinary incontinence, of particular interest is the implantation of autologous muscle stem cells into the sphincter area to strengthen and restore its function. In rats, injected muscle-derived SC led to the formation of myotubes and myofibers [12]. Moreover, autologous muscle-derived SCs were able to restore damaged urinary sphincter function with up to 80% of the initial closure pressure values [12]. For the treatment of anal incontinence, the regenerative effect on anal sphincter injuries has been examined with local injections of culture expanded skeletal myogenic cells in rats, rabbits, and dogs [13]. Autologous mesenchymal SC, as another potential candidate for cellular therapy, locally or intravenously injected in anal sphincters of rabbits, improves histological and functional regeneration due to transient paracrine stimulation of resident stem cells by the injected SC [13]. Despite the clinical evidence is very limited, the procedure appears to be safe and effective and represents a new potential strategy to treat anal incontinence caused by anal sphincter defects. However, safety and efficacy data of SCs for pelvic floor dysfunctions in clinical studies are scarce and limited to small populations. Moreover, while several narrative reviews are available about application of SCs for PFDs, there is lack of a systematic review summarizing and possibly pooling data of available clinical studies.

As a consequence, as the primary outcome, we aimed to define the state of the art of stem cell therapy for pelvic floor disorders in clinical trials, by systematically reviewing the available evidence. We intended to perform a meta-analysis of the available data but this was ultimately not possible with the heterogeneity of the data.

## Methods

### Study Protocol

This systematic review was conducted and reported according to both the PRISMA Statement for Reporting Systematic Reviews and Meta-Analysis [14] and the Meta-Analysis of Observational Studies in Epidemiology guidelines [15]. Study objectives, eligibility criteria, outcome definitions, search strategy, data extraction process, and method of study quality assessment were all defined in a protocol. The study protocol was registered in PROSPERO (CRD42020216551).

### Eligibility Criteria and Outcome Definition

Studies assessing the impact of stem cell therapy on pelvic floor disorders in clinical trials were included. Preclinical studies on animal models were not considered. Reviews, letters to editor, conference abstracts, book chapters, guidelines, Cochrane reviews, and expert opinions were excluded. Only papers in which cells were isolated/cultured and characterized as stem cells by the authors were considered.

### Data Source and Literature Search

To identify potentially eligible studies, we searched PubMed, Scopus, Cochrane Library, and ISI Web of Science (up to November 7, 2020), using EndNote × 8 (Clarivate Analytics, Philadelphia, USA). No language restrictions were applied. We used a combination of keywords and text words represented by “stem cells” and “prolapse,” “incontinence,” “pelvic floor,” “pelvic dysfunctions,” and “pelvic disorders.” An example of the complete search strategy used for the PubMed search is presented in Appendix S1. Two reviewers independently screened titles and abstracts of the records that were retrieved through the database searches. We also performed a manual search to include additional relevant articles, using the reference lists of key articles published in English. Both reviewers independently recommended studies for the full-text review. Full texts of records recommended by at least one reviewer were screened independently by the same two reviewers and assessed for inclusion in the systematic review. Disagreements between reviewers were solved by consensus.

### Data Extraction and Study Quality Evaluation

Data were extracted using a piloted form specifically designed for capturing information on study characteristics (sample size, outcomes, and considered variables). Data about outcome measures by the study were collected. Data for continuous variables were extracted as means and standard deviations; for categorical variables, data were extracted as absolute values. Data were extracted independently by two authors to ensure accuracy and consistency. We emailed the authors of excluded studies that we felt potentially may have relevant unpublished data. We received some answers, but no new dataset was obtained.

## Results

### Study Assessment

**Fig. 1:**
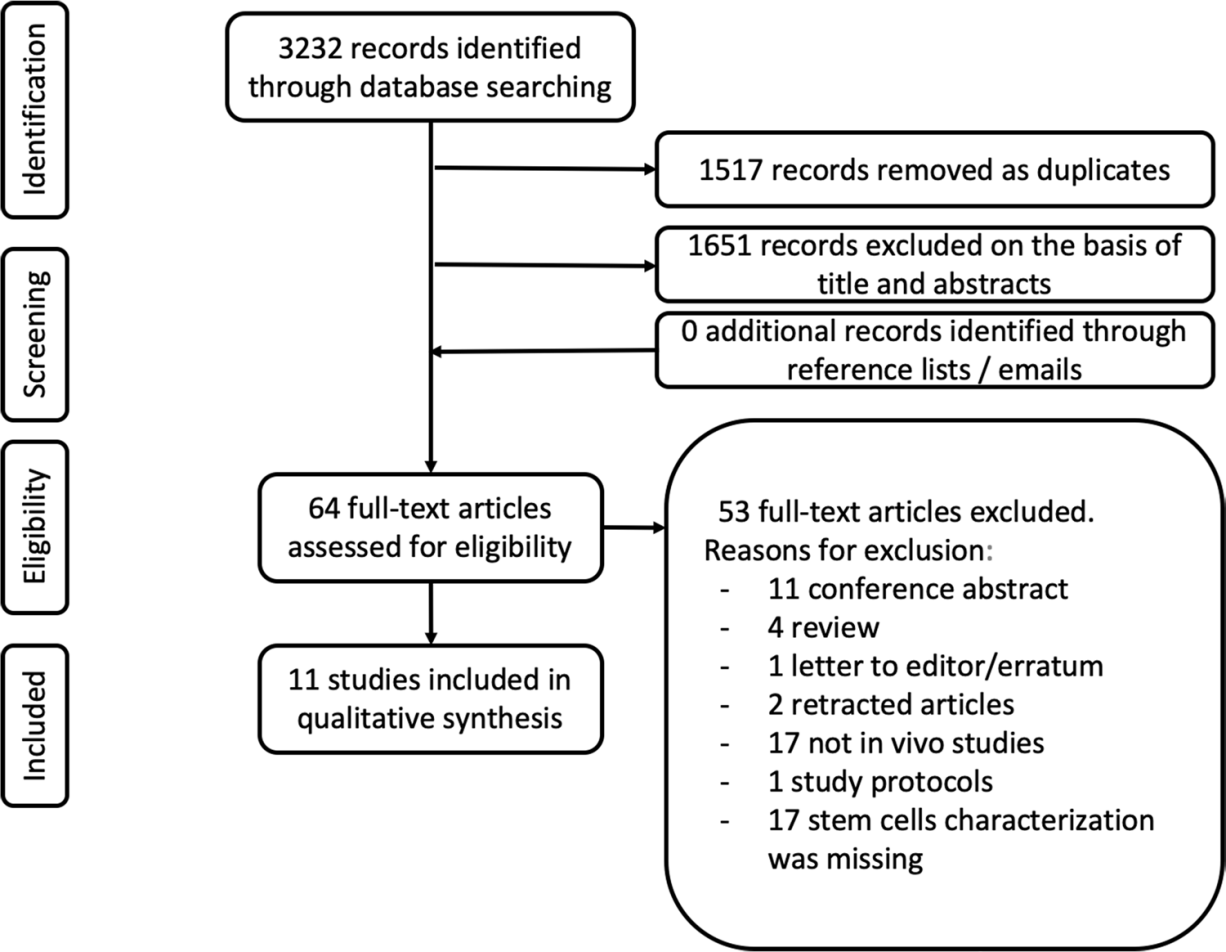
The electronic database search

The electronic database search provided a total of 3232 results (Fig. 1). After duplicate exclusion, there were 1715 citations left. Of them, 1651 were not relevant to the review based on title and abstract screening. Sixty-four studies were considered for full-text assessment, of which 53 were excluded for the following reasons. There were 4 reviews, 11 conference abstracts, 1 letter to the editors, 2 retracted papers, and 1 study protocol. Seventeen papers were excluded for being either in vitro or on animal models. Finally, 17 studies were excluded due to the lack of stem cells isolation/culture. None was excluded for languages other than English. No paper was added through reference list searching. Overall, 11 studies met the inclusion criteria and were incorporated into the final assessment [16–26]. Specifically, we found 7 papers dealing with stem cell therapy for stress urinary incontinence [16–22] and 4 with anal incontinence [23–26]. No papers concerning the use of SC for prolapse repair were retrieved. The main characteristics of these studies are listed in Tables 1 and 2 respectively. Different study designs resulted from the selection process, including prospective studies and randomized controlled trials. The studies included were very heterogeneous clinically. All the outcome measures proposed by the considered studies were analyzed.

**Table 1:** Study characteristics dealing with stem cell therapy for urinary incontinence

First author	Year	Ref	Country	Study design	Stem cell source	No. of stem cells	Application	No. of patients	Outcome measures	Results
Arjmand	2017	16	Iran	Prospective single-arm	Adipose-derived	1.8 × 10^6^	Trans- and periurethral	10	ICIQ-SF, 24-h pad test, Qmax	- Subjective outcomes: no significant improvement at 6 months - Objective outcomes: significant reduction at 6 months - Instrumental outcomes: Qmax significantly increased at 6 months
Carr	2008	17	Canada	Prospective single-arm	Muscle-derived	18–22 × 10^6^	Trans- and periurethral	8	24-h pad test, voiding diary	- 3/8 withdraw at 1 month because of no improvement - Objective outcomes: 5/8 improved with 1 achieving total continence at 12 months
Garcia-Arranz	2020	18	Spain	Prospective single-arm	Adipose-derived	40 × 10^6^	Transurethral	10	SF-36, ICIQ-SF, 24-h pad test, cough test, urodynamic evaluation	- Subjective outcomes: no significant improvement at 12 months - Objective outcomes: 6/10 were negative at 12 months at cough test; 5/10 patients improved at 12 months at 24-h pad test - Instrumental outcomes: no urinary incontinence in 5/10 patients at 12 months
Kuismanen	2014	19	Finland	Prospective single-arm	Adipose-derived	2.5–8.5 × 10^6^	Transurethral (+ collagen)	5	UISS, IIQ-7, UDI-6, and VAS, cough test, 24-h pad test, MUCP	- Subjective outcomes: 2/5 improved in all questionnaires - Objective outcomes: negative in 3/5 at 12 months at cough test; significant reduction in 2/3 patients with negative cough test at 12 months at 24-h pad test - Instrumental outcomes: no changes in MUCP at 12 months
Lee	2010	20	Korea	Prospective single-arm	Umbilical cord blood	4.3 × 10^6^	Transurethral	39	Patient Satisfaction Test, MUCP	- Patient satisfaction test: 26/39 showed more than 50% improvement at 12 months - In 10 patients with MUCP below 30 cmH_2_O before treatment, it increased by more than 30 after the procedure
Sharifiaghdas	2016	21	Iran	Prospective single-arm	Muscle-derived	38.6 × 10^6^	Transurethral	10	IIQ-7, 1-h pad test, MUCP	- Subjective outcomes: significant improvement at 12 months - Objective outcomes: mean improvement at 12 months (3/10 cured, 4/10 improved) - Instrumental outcomes: mean improvement at 12 months
Sharifiaghdas	2019	22	Iran	Prospective single-arm	Muscle-derived	50 × 10^6^	Transurethral	17	IIQ-7, UDI-6, cough test, 1-h pad test, MUCP, Qmax	- Subjective outcomes: improved in 10 complete responders at 24 months - Objective outcomes: cough test negative in 10 complete responders at 12 months; 1-h pad test negative in 10 complete responders at 12 months - Instrumental outcomes: no changes in MUCP at 24 months; Qmax significantly decreased in complete responders at 24 months

*ICIQ-SF*, International Consultation on Incontinence Questionnaire-Short Form; *IIQ-7*, Incontinence Impact Questionnaire-7; *MUCP*, maximum urethral closure pressure; *Qmax*, maximum flow rate; *SF-36*, Short Form Health Survey-36; *UDI-6*, Urinary Distress Inventory-6; *UISS*, Urinary Incontinence Severity Score; *VAS*, visual analogue scale

**Table 2:** Study characteristics dealing with stem cell therapy for anal incontinence

First author	Year	Ref	Country	Study design	Stem cell source	No. of stem cells	Application	No. of patients	Outcome measures	Results
De La Portilla	2020	23	Spain	Randomized triple-blinded trial	Adipose-derived	10^7^	Intrasphincteric injection	12F + 6 M	CCFIS, FIQL scale, anorectal physiology outcomes (maximal basal pressure, maximum voluntary contraction pressure, anal canal length, rectoanal reflex, rectal sensitivity threshold, and urgency)	CCFIS scores decreased over time, but not significantly differ between groups; rectum-anal reflex, rectal sensitivity threshold non-significantly reduced; urgency factor significantly reduced in both groups; no differences in manometry; FIQL significantly more responders in the placebo group
Frudinger	2018	24	Austria	Prospective single-arm	Muscle-derived	2.5 × 10^7^	Intrasphincteric injection	34F + 5 M	Incontinence diary, number of WIE, Wexner score, VAS, anorectal manometry, FIQL score, CGI score	At all post-implantation visits (to 1, 6, and 12 months), the number of WIE was substantially reduced; Wexner scores decrease statistically significant; VAS rapidly decreased; statistically significant higher FIQL at all visits; improvement of CGI score
Romaniszyn	2015	25	Poland	Prospective single-arm	Muscle-derived	10^8^	Intrasphincteric injection	9F + 1 M	Wexner score, FISI, manometry (BAP, SAP, HPZL), EMG, endorectal US	- 6 weeks fu: subjective improvement in questionnaires - 12 and 18 weeks fu: manometry and questionnaire improvement, EMG improvement - 12 months fu: manometry and EMG slightly deteriorated - At 18 weeks: subjective improvement in 6 patients - At 12 months: deterioration of continence in 2 out of 6 - Results include male
Sarveazad	2017	26	Iran	Randomized double-blind clinical trial	Adipose-derived	6 × 10^6^	During sphincteroplasty	14F + 4 M	Wexner scores, endorectal US, EMG	- 2 months fu: no differences in Wexner score between groups; significant difference in US; significant difference in EMG - No serious AE - Results include male

*AE*, adverse events; *CCFIS*, Cleveland Clinic Fecal Incontinence Score; *EMG*, electromyography; *F*, female; *FIQL*, Fecal Incontinence Quality of Life; *M*, male; *US*, ultrasound; *VAS*, visual analogue scale; *WIE*, weekly incontinence episodes

### Stem Cell Therapy for Stress Urinary Incontinence

To evaluate the impact of stem cell therapy on stress urinary incontinence, 7 prospective, single-arm studies were considered (Table 1) [16–22]. A total of 99 patients were considered. Three studies [16–18] enrolled patients with isolated stress urinary incontinence, regardless of the triggering cause; two studies included patients with isolated or mixed urinary incontinence [19, 20]; one study considered only patients affected by stress urinary incontinence secondary to intrinsic sphincter deficiency [21]; one study enrolled only patients with stress urinary incontinence secondary to urethral hypermobility [22]. The lower abdomen subcutaneous fat was the source of stem cells in three studies [16, 18, 19], while in other three studies, stem cells were harvested from the muscle tissue [17, 21, 22]; in one study, the stem cell source was the human cord blood [20]. Stem cell administration procedures included transurethral injection at the proximal urethra via cystoscope, periurethral injection through the skin, and transvaginal periurethral injection. The number of injected cells ranged from 1.8 × 10^6^ to 50 × 10^6^. The largest number of cells was injected when stem cells were muscle-derived. The volume of injected cells was less than 10 ml in all the studies.

No serious adverse effects were reported after SC injection. Reported minor adverse events were 2 cases of hematoma formation during adipose tissue collection [18, 19], 3 cases of dysuria that spontaneously resolved a few days after cell injection [16, 18, 19], and 2 cases of urinary tract infection [21]. In one study, 2 patients withdrew due to pain during the procedure, although local anesthesia with lidocaine was administered prior to cystoscopic injection at 4 and 8 o’clock in the urethra [20].

Both outcome measures and time points were very heterogeneous in the considered studies. In consideration of the given limitations, data pooling was not possible.

Different objective outcomes of treatment success were considered, including cough stress test [18, 19, 22], 24-h pad weight [16–19], 1-h pad test [21, 22], and voiding diary [17]. A negative cough test at 12 months was reported by Kuismanen et al. [19] in 3 out of 5 patients (60%) and in 6 out of 10 patients (60%) by Garcia-Arranz et al. [18]. Sharifiaghdas et al. [22] described a negative cough test at 12 months in 10 out of 17 patients (59%), but recurrence of SUI after 24 months occurred in 5 of the complete responders (50%). A significant reduction in the 24-h or 1-h pad weight test at the final follow-up visit (from 24 weeks [16] to 36 months [21]) was reported by all the studies [16–19, 21, 22].

A significant improvement in subjective outcomes was reported by 4 studies [19–22], while 2 studies [16, 18] failed to demonstrate better quality of life in treated patients. Kuismanen et al. [19] reported an improvement in all the questionnaires (UISS, IIQ-7, UDI-6, VAS) in 2 out of 3 patients (67%) with a negative cough test at 12 months. Lee et al. [20] reported that 26 out of 39 patients (67%) had a more than 50% improvement in patient satisfaction test score at 12 months. A significant improvement compared to baseline in IIQ-7 and UDI-6 scores was described by Sharifiagdas et al. in both their studies at 12 and 24 months [21, 22]. Otherwise, Arymand et al. [16] and Garcia-Arranz et al. [18] failed to prove any improvement respectively at 6 months and 12 months. Carr et al. [17] stated that 3 out of 8 patients (38%) withdrew from the study because of no subjective improvement at 1 month.

The outcomes regarding urodynamic test parameters turned out to be highly variable. No changes in maximal urethral closing pressure (MUCP) were reported by Kuismanen et al. [19] and Sharifiagdas et al. [22] respectively at 12 and 24 months after the procedure. On the contrary, an improvement in mean MUCP values was described by Sharifiagdas et al. [21] at 12 months. Similarly, Lee et al. [20] described a significant increase in MUCP values after the procedure in patients with a baseline MUCP below 30 cmH_2_O. Lastly, Garcia-Arranz et al. [18] reported the absence of urinary incontinence at urodynamic evaluation in 5 out of 10 patients (50%) at 12 months.

### Stem Cell Therapy for Anal Incontinence

In evaluating the impact of stem cell therapy on anal incontinence, 4 studies were analyzed (Table 2) [23–26]. Studies’ design included two prospective single-arm studies [24, 25] and two randomized controlled trials [23, 26]. In total, 66 patients underwent stem cell therapy in the considered studies, including 10 men. Considered forms of anal incontinence included passive, urgency incontinence, and soiling. Stem cells were harvested from the adipose tissue [23, 26] or the muscular tissue [24, 25]. Stem cell administration procedures included direct injection in the external or in the internal anal sphincter, percutaneously or during sphincteroplasty. The number of injected cells ranged from 6 × 10^6^ to 10^8^. No severe adverse effects were reported after SC injection. De la Portilla et al. had one case (6.3%) of hematoma in the adipose SC harvest site, but none associated with the injection procedure [23]. Sarveazad et al. reported one case (11.1%) of erythema in the surgical site [26]. No other complications were reported by the remaining studies [24, 25].

Both outcome measures and time points were very heterogeneous in the considered studies. In consideration of the given limitations, data pooling was not possible.

Objective outcomes resulted inconsistent [23, 24]. Frudinger et al., in a population of 34 female patients who underwent external anal sphincter, observed that SC injection had a significant decrease of weekly incontinence episodes at all considered time points (V2 = days 1 to 28; V3 = days 140 to 168; V4 = days 337 to 365) compared to baseline (− 9.2 at V2; − 10.6 at V3; − 11.0 at V4) [24]. Conversely, de la Portilla et al. did not find any significant decrease in the number of episodes of incontinence in the stem cell group (8 patients), either compared to baseline or placebo group at any considered time point (4, 12, 24, and 48 weeks) [23].

Improvements in subjective outcomes were reported by both the single-arm prospective studies evaluating autologous muscle-derived stem cell implantation. Romaniszyn et al. reported subjective improvement both in Wexner and FISI (Fecal Incontinence Severity Index) scores at 6, 12, and 18 weeks compared to baseline in all 9 patients (100%) who completed the full 12-month follow-up. Twelve months after implantation, 4 patients (44%) continued to have satisfactory results with a reduction in frequency and intensity [25]. Similarly, Frudinger et al. evaluated patients’ quality of life on the Fecal Incontinence Quality of Life (FIQL) scale demonstrating higher scores at all considered time points compared to baseline. Moreover, all pre-post differences from V0 to every time point up to days 337 to 365 reached significance. Similarly, perceived severity of fecal incontinence on a VAS scale rapidly decreased after treatment. At the end of the trial (V4 = days 337 to 365), the 7-point Clinical Global Impression (CGI) scale was submitted to assess the rate of treatment-induced changes showing improvement for all patients except for one (97.4%) for whom “no change” was reported [24]. However, the impact of stem cell injection in the controlled studies resulted less clear. Sarveazad et al. showed that injection of human adipose-derived stem cells in fecal incontinence repair surgery (9 sphincteroplasty) can achieve an acceptable improvement in Wexner scores at 2 months follow-up but, when compared with the placebo group, the efficacy of cell therapy—as well as conventional therapy—was not significantly different [26]. Similarly, subjective effectiveness assessed through the Cleveland Clinic Fecal Incontinence Score (CCFIS) scale by de la Portilla et al. did not significantly differ between stem cells and placebo groups, and no time interaction was found, though the CCFIS score reduction was higher in the study group than the placebo. However, there were significantly more responders in the placebo group for all subcategories of the FIQL scale [23].

Anal manometry evaluation showed inconsistent results. Frudinger et al. demonstrated a significant improvement in anal manometry findings in terms of an increase in the functional length of the anal canal of 11 mm compared to baseline, along with an increase in the first desire volume of 16 mm [24]. Romaniszyn et al. reported an increase in mean resting pressure, squeeze anal pressure, and high-pressure zone length at 18 weeks and 12 months compared to baseline [25]. However, they recorded a deterioration of manometric parameters from 18-week to 12-month time points. On the contrary, in a randomized controlled trial, de la Portilla et al. did not find any benefit in terms of manometric findings in the SC injection group compared to placebo administration [23].

Endoanal ultrasound evaluation demonstrated minimal to no benefits. In one study, the amount of muscle in the repair site calculated by ultrasound-dedicated software was found to be increased in patients who received SC injection compared to placebo (+ 7.9%; *p* = 0.02) [26]. On the contrary, other two studies did not report any significant difference in ultrasound findings [23–25].

Electromyography evaluation after SC injection was performed by two studies that demonstrated a significant improvement in the recorded electrical activity [25, 26]. Romaniszyn et al. reported an increase in signal amplitude, detecting an elevated number of propagating potentials at 18 weeks after SC injection, which declined but was still significantly better than baseline at 12 months [25]. Similarly, Sarveazad et al. demonstrated a significantly higher EMG activity at 2 months after SC injection compared to controls [26].

## Discussion

### Main Findings of the Systematic Review

Stem cells and regenerative medicine represent a promising alternative option for the treatment of PFDs. However, safety and efficacy data of SCs for pelvic floor dysfunctions in clinical studies are scarce and limited to small populations. Our systematic review identified seven and four clinical studies regarding stem cell therapy for stress urinary and anal incontinence respectively, while none was found for pelvic organ prolapse. Overall, the number of patients who underwent SC therapy was limited. Stem cell injection resulted in a safe procedure, with few mild adverse side effects, mostly related to harvesting sites. Moreover, due to great heterogeneity in terms of study design, inclusion criteria, stem cell harvesting/delivery, outcome measures, and time points, data pooling was not possible. However, reported outcomes were contrasting, and a clear beneficial impact of SC treatment for the treatment of pelvic floor disorders could not be demonstrated.

### Stem Cell Therapy for Stress Urinary Incontinence

Seven prospective, single-arm studies were analyzed to evaluate the efficacy and safety of stem cell therapy on stress urinary incontinence [16–22]. Regarding stem cell injection safety, only minor adverse events were reported by all the studies and, although the small number of patients was included, it is possible to consider this procedure safe, at least in the short term. On the other hand, results on efficacy in the different studies are very heterogeneous. When considering subjective or objective outcomes, trials using adipose-derived stem cells demonstrated only a mild or no improvement [16, 18, 19]. Differently, studies with muscle-derived stem cells [17, 21, 22] or with human cord blood stem cells [20] reported a higher benefit in terms of patient satisfaction. Regarding the instrumental outcomes, heterogeneity between studies was too significant to draw some conclusions. In addition to different cell lines, differences between studies in sample size, number of cells injected, and duration of follow-up account for discrepancies in results and their lack of comparability.

### Stem Cell Therapy for Anal Incontinence

In evaluating the impact of stem cell therapy on anal incontinence, 4 studies were analyzed including two prospective single-arm studies [24, 25] and two randomized controlled trials [23, 26]. Results after SC injection reported by these papers resulted in inconsistent and limited benefits, for all considered outcomes, including objective, subjective, and instrumental ones. This was particularly true for both randomized controlled trials, who found either no benefits [23] or a mild increase in calculated muscle volume and EMG activity [26] after adipose-derived stem cell injection, when compared to placebo therapy. More promising results were found by the studies involving the use of muscle-derived stem cells in terms of objective, subjective, and instrumental functional findings [24, 25]. This might indicate a role in the choice of the most appropriate source of stem cells for the treatment of sphincter deficits. However, the lack of a control group in these papers limits the possibility to draw any conclusion.

### Strength, Limitations, and Future Perspectives

The major strength of our analysis is giving for the first time a systematic presentation of prospective studies in the expanding and high-interest field of SCs for PFDs. However, there are certain limitations inherent to this systematic review. Studies were very heterogeneous in terms of study designs, indications for treatment, populations considered (sometimes including also male patients), stem cell sources, procedures performed to deliver SC, outcome measures, and time points, which leads to the fact that we cannot compare/merge data. Moreover, given the experimental use of SC in clinical settings, the sample size resulted in small populations, which may be underpowered to detect changes in the considered outcomes.

Stem cell therapy represents a fascinating and promising option for the treatment of pelvic floor disorders. However, up to date, there are limited experiences with very heterogeneous methods and processes that need better definition and standardization before any conclusion can be drawn. Cell harvesting, isolation, expansion, and implantation are complex and expensive procedures. Depending on the source (adipose tissue, bone marrow, or skeletal muscle), the harvesting process may also expose the patient to additional risk of morbidity at the harvest site. Moreover, the optimal dose of SCs is not well defined and may vary according to the type of cell and the aim of the treatment. Another key point is post-implantation differentiation. Aberrant differentiation may lead to non-functional tissue, and paracrine effects of the microenvironments of the tissue—as those exerted by chemokines, secretomes, cytokines, growth and angiogenic factors—are important determinants of cell fate [F]. As a consequence, future studies should focus on optimal source, dose and delivery route, to minimize harvesting morbidity and costs, avoid abnormal differentiation, and optimize therapeutic efficacy.

## Conclusions

In conclusion, our systematic review found the currently studied SC therapies for pelvic floor disorders to be safe procedures. However, we did not find clear evidence for a beneficial impact of SC treatment for the treatment of pelvic floor disorders. The role of SCs in the treatment of pelvic floor disorders needs to be further evaluated in larger targeted studies with control arms before any conclusions can be made.

## Supplementary Information

Below is the link to the electronic supplementary material.

Supplementary file1 (DOCX 13 KB)

Supplementary file2 (DOCX 17 KB)

Supplementary file3 (DOCX 16 KB)

## Data Availability

The datasets analyzed during the current study are available from the corresponding author on reasonable request.
