# Supplementary material for: Stem Cells in Clinical Trials for Pelvic Floor Disorders: a Systematic Literature Review
Source: Reprod Sci. 2021 Oct 1;29(6):1710–20. doi: 10.1007/s43032-021-00745-6 (PMC9110489; doi:10.1007/s43032-021-00745-6)
Supplement: Supplementary file 1 — Supplementary file1 (DOCX 13 KB) [file 43032_2021_745_MOESM1_ESM.docx]

**Supplementary Table 1**. Search strategy.

|  | Stem cells AND prolapse 72 |
| --- | --- |
| [#1](http://www.ncbi.nlm.nih.gov/pubmed/advanced) | ("stem cells"[MeSH Terms] OR ("stem"[All Fields] AND "cells"[All Fields]) OR "stem cells"[All Fields]) AND ("prolapse"[MeSH Terms] OR "prolapse"[All Fields] OR "prolapses"[All Fields] OR "prolapsed"[All Fields] OR "prolapsing"[All Fields]) |
|  | Stem cells AND incontinence 399 |
| #2 | ("stem cells"[MeSH Terms] OR ("stem"[All Fields] AND "cells"[All Fields]) OR "stem cells"[All Fields]) AND ("incontinance"[All Fields] OR "incontinence"[All Fields] OR "incontinences"[All Fields] OR "incontinency"[All Fields] OR "incontinent"[All Fields] OR "incontinents"[All Fields]) |
|  | Stem cells AND pelvic floor 79 |
| #3 | ("stem cells"[MeSH Terms] OR ("stem"[All Fields] AND "cells"[All Fields]) OR "stem cells"[All Fields]) AND ("pelvic floor"[MeSH Terms] OR ("pelvic"[All Fields] AND "floor"[All Fields]) OR "pelvic floor"[All Fields]) |
|  | Stem cells AND pelvic disorders 198 |
| #4 | ("stem cells"[MeSH Terms] OR ("stem"[All Fields] AND "cells"[All Fields]) OR "stem cells"[All Fields]) AND (("pelvics"[All Fields] OR "pelvis"[MeSH Terms] OR "pelvis"[All Fields] OR "pelvic"[All Fields]) AND ("disease"[MeSH Terms] OR "disease"[All Fields] OR "disorder"[All Fields] OR "disorders"[All Fields] OR "disorder s"[All Fields] OR "disordes"[All Fields])) |
|  | Stem cells AND pelvic dysfunctions 104 |
| #5 | ("stem cells"[MeSH Terms] OR ("stem"[All Fields] AND "cells"[All Fields]) OR "stem cells"[All Fields]) AND (("pelvics"[All Fields] OR "pelvis"[MeSH Terms] OR "pelvis"[All Fields] OR "pelvic"[All Fields]) AND ("dysfunctional"[All Fields] OR "dysfunctionals"[All Fields] OR "dysfunctioning"[All Fields] OR "dysfunctions"[All Fields] OR "physiopathology"[MeSH Subheading] OR "physiopathology"[All Fields] OR "dysfunction"[All Fields])) |
