# Supplementary material for: Stem Cells in Clinical Trials for Pelvic Floor Disorders: a Systematic Literature Review
Source: Reprod Sci. 2021 Oct 1;29(6):1710–20. doi: 10.1007/s43032-021-00745-6 (PMC9110489; doi:10.1007/s43032-021-00745-6)
Supplement: Supplementary file 3 — Supplementary file3 (DOCX 16 KB) [file 43032_2021_745_MOESM3_ESM.docx]

| **First Author** | **Year** | **Ref** | **Topic** | **Source** | **Potential** | **Origin** |
| --- | --- | --- | --- | --- | --- | --- |
|  |  |  |  |  |  |  |
| Arjmand | 2017 | 16 | UI | Adipose-derived stem cells | Peripheral multipotent | Autologous |
| Carr | 2008 | 17 | UI | Muscle-derived stem cell | Unipotent myogenic precursor cells | Autologous |
| Garcia | 2020 | 18 | UI | Adipose-derived stem cells | Peripheral multipotent | Autologous |
| Kuismanen | 2014 | 19 | UI | Adipose-derived stem cells | Peripheral multipotent | Autologous |
| Lee | 2010 | 20 | UI | Cord blood stem cell | Multipotent | Homologous |
| Sharifiaghdas | 2016 | 21 | UI | Skeletal muscle-derived cells | Unipotent myogenic precursor cells | Autologous |
| Sharifiaghdas | 2019 | 22 | UI | Skeletal muscle-derived cells | Unipotent myogenic precursor cells | Autologous |
| De La Portilla | 2020 | 23 | AI | Adipose-derived stem cells | Peripheral multipotent | Autologous |
| Frudinger | 2018 | 24 | AI | Skeletal muscle-derived cells | Unipotent myogenic precursor cells | Autologous |
| Romaniszyn | 2015 | 25 | AI | Skeletal muscle-derived cells | Unipotent myogenic precursor cells | Autologous |
| Sarveazad | 2017 | 26 | AI | Adipose-derived stem cells | Peripheral multipotent | Homologous |
